# Toll-like receptor-4 is upregulated in plaque debris of patients with acute coronary syndrome more than Toll-like receptor-2

**DOI:** 10.1007/s00380-014-0565-9

**Published:** 2014-09-02

**Authors:** Shinji Satoh, Ryoko Yada, Hiroko Inoue, Soichiro Omura, Emiko Ejima, Takahiro Mori, Katsuhiko Takenaka, Natsumi Kawamura, Kotaro Numaguchi, Etsuo Mori, Akemi Asoh, Toshihiro Nakamura, Koji Hiyamuta

**Affiliations:** 1 Division of Cardiology, National Hospital Organization Kyushu Medical Center, 1-8-1 Jigyohama, Chuo-ku, Fukuoka, 810-8563 Japan; 2 Clinical Research Institute, National Hospital Organization Kyushu Medical Center, 1-8-1 Jigyohama, Chuo-ku, Fukuoka, 810-8563 Japan

**Keywords:** Acute coronary syndrome, Atherogenesis, Distal protection, Inflammation, Toll-like receptor

## Abstract

Atherosclerosis is a dise ase characterized by inflammation in the arterial wall. Atherogenesis is dependent on the innate immune response involving activation of Toll-like receptors (TLRs) and the expression of inflammatory proteins, those may lead to acute coronary syndrome (ACS). We investigated the expression level of TLR-4 in ACS, as compared with TLR-2 and patients with stable angina. Fifty-eight consecutive patients who underwent primary percutaneous coronary intervention (PCI, *n* = 29) because of ACS and elective PCI (*n* = 29) because of stable angina using a filter-device distal protection device system were prospectively analyzed. mRNA levels of TLR-2 and TLR-4 in debris containing various inflammatory tissues entrapped in the filter device were altogether analyzed using real-time PCR. There were no significant differences in age, sex distribution, between stable angina and ACS groups. TLR-4 expression levels were higher in patients with ACS than in patients with stable angina. TLR-4 might play a more important role than TLR-2 in atherogenesis, especially in ACS.

## Introduction

Atherosclerosis is a disease characterized by inflammation in the arterial wall [1]. Inflammation has been shown to play an important role not only in the formation of atheromatous plaque, but also in the progression of stable plaque to vulnerable one causing acute coronary syndrome (ACS), such as unstable angina and acute myocardial infarction [2]. Several lines of evidence have suggested that Toll-like receptors (TLRs) are the most characterized receptors for innate immune responses, and they play a role in promoting atherogenesis and cardiovascular diseases via expression of inflammatory proteins [3–5].

Among them, TLR-2, -3, and -4 were reported to play pivotal roles in myocardial injury after ischemic events [6–8] and ACS [9]. It thus would be interesting to investigate how much TLRs expression is in atheromatous plaque. We herein analyzed the expression of TLRs in patients with ACS arising from vulnerable plaque, as compared with that in patients with stable ischemic patients.

## Methods

This was a prospective observational study in 58 patients who underwent emergent primary percutaneous coronary intervention (PCI) because of ACS, that is, acute ST-segment elevation myocardial infarction (STEMI) and unstable angina (*n* = 29), or elective PCI because of stable angina (*n* = 29) both using a filter-device distal protection device system were enrolled.

**Fig. 1:**
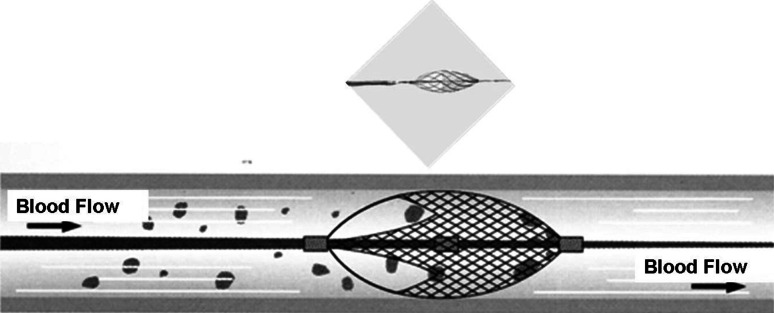
Photo and schema of the Filtrap

The latest guidelines of ACC/AHA [10] were used to select patients with ACS that presented acute ischemic symptoms lasting ≥30 min and ischemic electrocardiogram (ECG) changes, and/or positive cardiac biomarkers (troponin-T and/or creatine kinase-MB). Ischemic ECG changes were defined as transient ST-segment elevation or depression, and newly appeared negative U waves. STEMI was defined as persistent chest pain lasting for ≧30 min associated with new ST-segment elevation in ≧2 contiguous leads with a cutoff point of ≧0.2 mV. Myocardial damage was confirmed by elevation of the levels of CK-MB (≧2-fold the upper limit of the normal range) or troponin-T during the patient’s clinical course. Coronary angiography revealed at least one culprit lesion defined as an occluded coronary artery or an artery with 90–99 % stenosis based on a visual estimate and the angiographic appearance of the thrombus. A total of 29 consecutive ACS patients performed the emergent primary PCI with distal protection using a filter-device system (Filtrap^®^, Fig. 1) based on the operator’s decision and anatomical accessibility; that is, vessels were not tortuous and the diameter ≥3 mm. Another group consisted of 29 consecutive patients with stable angina performed the elective PCI also using the filter-device system. Stable angina was defined as the presence of chest pain on an almost fixed level of effort unchanged over the previous 1 month, a positive stress test by ECG and/or thallium myocardial perfusion scintigraphy, and significant vessel stenosis ≧75 % in at least 1 major coronary artery on selective coronary angiography.

The patients underwent coronary angiography and PCI according to standard techniques using a femoral or radial/brachial approach, followed by the intra-arterial administration of 100 IU/kg of heparin [11]. We applied the distal protection device in primary or elective PCI to prevent distal emboli and subsequent slow/no flow which is caused by protruded plaque contents after balloon dilatation and stent implantation. In the PCI with distal protection, the distal protection procedure was similarly performed as previously reported by Maier et al. [12] We crossed the culprit lesion with a filter-device system (Filtrap^®^) after crossing the lesion with a conventional guidewire. After crossing the culprit lesion, the filter device was inflated for distal protection of the culprit artery. A 6-French Eliminate aspiration catheter (Clinical Supply Co., Ltd., Gifu, Japan) was inserted over the guidewire, and two to three passages of aspiration with a syringe via continuous aspiration from the distal end at the site of the filter to the upper border of the culprit lesion were performed. Subsequently, PCI was carried out following predilatation using a slightly undersized balloon when necessary for stent delivery, followed by single or multiple stenting. After this procedure, the filter device was deflated and retrieved. In all patients, following restoration of the antegrade coronary flow, same doses of intracoronary nitrates were administered to both groups in order to ensure maximal epicardial vasodilation in order to determine the size and length of the stent and to facilitate stent placement. All placed stents were bare-metal or drug-eluting stents. When a thrombolysis in myocardial infarction (TIMI) flow grade of 2 or 3 was achieved in STEMI patients, no further PCI was performed. Patients with a culprit lesion in the left main trunk and/or cardiogenic shock were excluded. Patients with autoimmune or inflammatory diseases (including patients treated with steroids and/or non-steroidal anti-inflammatory drugs), active infection, malignant diseases or liver cirrhosis were also excluded.

The restoration of coronary blood flow was assessed by a myocardial blush grade (MBG) as previously defined by Van’t Hof et al. [13] and that of a TIMI flow grade as previously described [14]. Angiographic evidence of thrombi was assessed according to the criteria summarized by Mabin et al. [15].

All patients were administered pharmacological treatment before PCI including the administration of aspirin (100 mg), heparin (5,000 IU) and clopidogrel (a loading dose of 300 mg). The standard therapy after PCI included aspirin, clopidogrel, beta-blockers, lipid-lowering agents such as statins, and angiotensin-converting enzyme inhibitors or angiotensin II receptor blockers, according to the current guidelines [10].

Blood tests were performed on admission under fasting or casual conditions. The blood samples were obtained on admission and every eight hours serially for 3 days in both groups.

Written informed consent for cardiac catheterization and data utilization was obtained from each patient, and the study protocol conformed to the ethical guidelines of the 1975 Declaration of Helsinki, as reflected in a priori approval by our institution’s human research committee.

### Real-time polymerase chain reaction (RT-PCR)

mRNA was extracted from the plaque debris entrapped in the filter device, and the debris contained lipid, macrophages, T- and B-lymphocytes, and other inflammatory cells. mRNA levels of TLR2 and TLR4 in debris entrapped in the filter device were collectively analyzed using real-time polymerase chain reaction (RT-PCR) using the QIAGEN 205313 Kit (QIAGEN, Tokyo, Japan) with the reagent of TAKARA RR081 (TaKaRa Biomedicals, Tokyo, Japan) according to the manufacturer’s specifications.

mRNA (51 bp) of Homo sapiens Toll-like receptor 2 (TLR-2: accession number NM 003264), was amplified by RT-PCR with specific primers 5′-GCCAGGCGGCTGCTC-3′ (sense) and 5′-TTGCAACACCAAACACTGGG-3′ (antisense), and that (64 bp) of Homo sapiens Toll-like receptor 4 (TLR-4), transcript variant 1 (accession number NM 138554) was amplified by RT-PCR with specific primers 5′-CAGAACTGCAGGTGCTGGATT-3′ (sense) and 5′-TGATATGCCCCATCTTCAATTG-3′ (antisense). The amounts of TLR-2 and TLR-4 were normalized to that of Homo sapiens beta-2-microglobulin (B2 M, 94 bp: accession number NM 004048.2) with specific primers 5′-CGGGCATTCCTGAAGCTGA-3′ (sense) and 5′-GGATGGATGAAACCCAGACACATAG-3′ (antisense) [TaKaRa ID: HA067806].

The relative expression levels of these TLR-2 and TLR-4 normalized to those of human β2-microglobulin were compared in the two patients groups.

### Statistical analysis

Because the continuous parameters in the present study were found to exhibit a non-normal distribution, the data are expressed as the median [interquartile range] for continuous variables and numbers and percentages for categorical variables. We therefore performed the statistical analysis using the nonparametric method. Comparisons of continuous variables between two groups were performed using the Mann–Whitney test or Wilcoxon signed-rank test. The Chi-square test or Fisher’s exact test were used to compare categorical variables. A two-tailed *p* value of <0.05 was considered to be statistically significant. All statistical analyses were performed using the GraphPad Prism^®^ 5.0 (GraphPad Software, Inc., San Diego, USA) software packages.

## Results

The baseline demographic and clinical characteristics of the patients are summarized in Table 1. There were no significant differences in age, the distribution of gender, coronary risk factors except the incidence of hypertension, the renal function as estimated by estimated glomerular filtration rate (eGFR) or pre-hospital medications, except that angiotensin-converting enzyme inhibitors/angiotensin II receptor blockers and calcium channel blockers had been more frequently prescribed in the ACS patients. The serum levels of low-density lipoprotein cholesterol (LDL) and C-reactive protein were higher in the ACS patients.

**Table 1:** Comparison of demographic and clinical characteristics of patients on admission

Variables	Elective	Emergent (ACS)	*p* value
*n*	29	29	
Age, years	70 [62–76]	67 [49–76]	0.3345
Males, *n* (%)	22 (75.8)	26 (89.7)	0.2973
Risk factors, *n* (%)			
Hypertension	27 (93.1)	18 (62.1)	0.0099
Dyslipidemia	22 (75.9)	25 (86.2)	0.5045
Diabetes mellitus	8 (27.6)	8 (27.6)	1.000
Smoking	6 (20.7)	7 (24.1)	1.000
Biochemical data			
LDL cholesterol, mg/dL	89 [70–106]	111 [96–131]	0.0003
HDL cholesterol, mg/dL	46 [36–55]	44 [39–55]	0.7973
HemoglobinA1c, %	5.8 [5.5–6.3]	5.6 [5.4–6.5]	0.7612
eGFR, mL/min/1.73 m^2^	64.6 [41.1–78.5]	70.8 [58.5–94.4]	0.1395
C-reactive protein, mg/dL	0.130 [0.055–0.56]	0.28 [0.14–2.255]	0.0192
Pre-hospital medication, *n* (%)			
Statins	25 (86.2)	24 (82.8)	1.000
Beta-blockers	15 (51.7)	17 (58.6)	0.7921
Angiotensin-converting enzyme inhibitors/angiotensin II receptor blockers	13 (44.8)	22 (75.9)	0.0307
Calcium channel blockers	10 (34.5)	20 (69.0)	0.0173
Nitrates	4 (13.8)	1 (3.4)	0.3525

The data are expressed as the median [interquartile range] for continuous variables and numbers and percentages for categorical variables

*eGFR* estimated glomerular filtration rate, *LDL* low-density lipoprotein, *HDL* high density lipoprotein

**Fig. 2:**
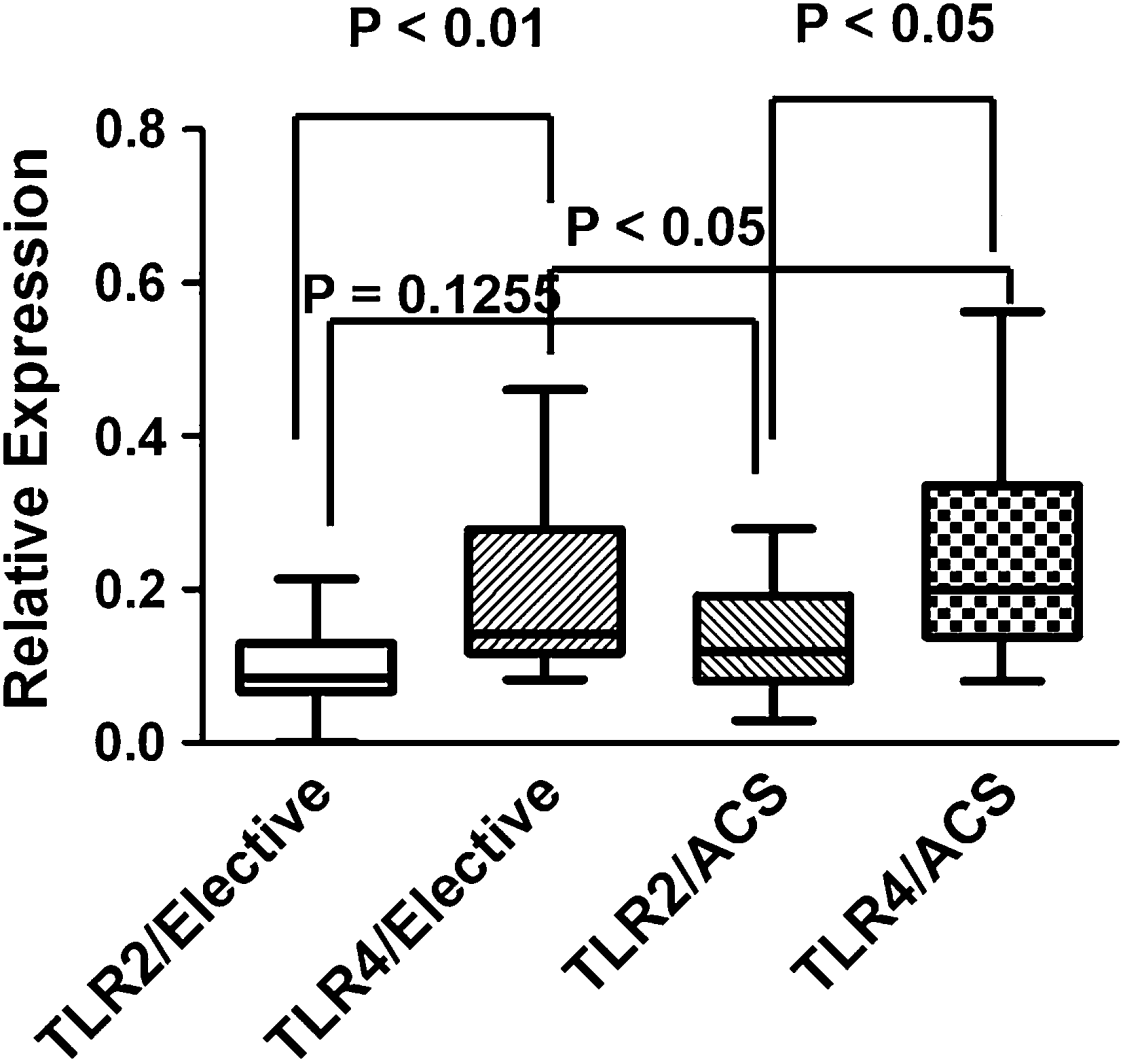
Comparison of the expression of TLR2 and TLR4

The mRNA expression levels of TLR-4 in the plaque debris were significantly higher than those of TLR2 in all enrolled patients, and they were significantly higher in patients with ACS than with stable angina. The expression levels of TLR-2 tended to be higher in patients with ACS than in stable angina although this did not reach statistical significance (*p* = 0.1255, Fig. 2).

## Discussion

The present study demonstrated while the expression levels of TLR-4 were higher than those of TLR-2 in all patients, those of TLR-4 were higher in patients with ACS than in stable angina. TLRs might be involved in the pathogenesis of ACS.

Many lines of evidence have been suggested that Toll-like receptors, which were discovered as a key receptor for innate and acquired immunity, play an important role not only in the formation of atheromatous plaque, but also in the progression of stable plaque to vulnerable one causing ACS [3, 4]. TLR-2 and TLR-4, subfamilies of TLRs, were reported to play pivotal roles in reperfusion myocardial injury after ischemic events [7, 16, 17]. TLR-4 may be involved not only in atherogenesis, but also in the progression of atheromatous plaque to vulnerable plaque causing ACS [9].

The following study limitations are noted. First, this study was a small-sized non-randomized observational study encompassing a selection bias. However, the patients’ clinical backgrounds and the distribution of coronary risk factors were similar. Second, differences in pre-hospital medications and levels of LDL cholesterol and C-reactive protein might affect the expression levels of TLRs.

In conclusion, TLRs may be involved in atherogenesis. Particularly, TLR-4 might play a more important role than TLR-2 in plaque instability, especially in ACS.
